# Theory of Mind Is Impaired in Mild to Moderate Huntington’s Disease Independently from Global Cognitive Functioning

**DOI:** 10.3389/fpsyg.2017.00080

**Published:** 2017-01-26

**Authors:** Giovanna Lagravinese, Laura Avanzino, Alessia Raffo De Ferrari, Roberta Marchese, Carlo Serrati, Paola Mandich, Giovanni Abbruzzese, Elisa Pelosin

**Affiliations:** ^1^Section of Human Physiology and Centro Polifunzionale di Scienze Motorie, Department of Experimental Medicine, University of GenoaGenoa, Italy; ^2^Department of Neuroscience, Rehabilitation, Ophthalmology, Genetics and Maternal Child Health, University of GenoaGenoa, Italy; ^3^Department of Neurology, IRCCS San Martino Hospital-ISTGenoa, Italy

**Keywords:** Huntington’s disease, theory of mind, reading the mind in the eyes test, social cognition, cognitive impairment

## Abstract

Affective “Theory of Mind” (ToM) is the specific ability to represent own and others’ emotional states and feelings. Previous studies examined affective ToM ability in patients with Huntington’s disease (HD), using the “Reading the Mind in the Eyes test” (RMET). Results were consistent in showing difficulties in inferring complex mental states from photographs of people even in the early stage of HD. However, there has been no agreement as to whether or not cognitive impairments in HD population might have contributed to poor performance on the RMET test. The aim of the present study was to assess whether the affective ToM ability was impaired in the mild to moderate stages of HD, and whether there was an association between compromised ToM ability and the presence of cognitive impairment. We evaluated ToM by means of RMET and global cognitive functioning by means of the MoCA questionnaire in 15 HD patients and 15 healthy subjects (HS). Both groups were matched for age and level of education. Our study showed that the ability to judge a person’s mental states from a picture of their eyes was impaired in HD patients compared to normal population. Indeed, HD subjects gave the 34% of correct responses on RMET, whereas healthy control subjects’ percentage of correct responses was 71%. Furthermore, this impairment was not correlated with global cognitive functioning except for the visuospatial task. These results show that RMET might represent a valid instrument to assess affective ToM ability in HD patients in the mild to moderate stages of the disease, independently from their cognitive status. Since it is known that HD patients, in addition to motor symptoms, suffer from cognitive deficits, including memory and executive impairments, it is important to have an instrument, which is not influenced by cognitive abilities. It is possible therefore to use RMET to assess important aspects of HD patients such as their ability to recognize others’ emotions and feelings even when patients suffer from cognitive decline.

## Introduction

Huntington’s disease (HD) is a hereditary neurodegenerative disorder, caused by an expansion of CAG triplet repeats in the *Huntingtin* gene on chromosome 4. Clinically, HD is characterized by motor symptoms (chorea, slowed saccadic eye movement, and abnormal posturing) as well as by impairment of cognitive abilities. Robust evidence demonstrated that cognitive decline might precede the onset of motor symptoms being associated with meaningful outcomes, including loss of accustomed work and poor quality of life (Paulsen, 2011). HD patients present a broad range of psychological disturbances such as cognitive rigidity, mood disturbances, lack of empathy and breakdowns of social relationships that might also manifest even before the onset of motor dysfunction (Marvel and Paradiso, 2004). In accordance, recent studies indicated that patients with HD present deficits in “social cognition” (Bora et al., 2016; Maurage et al., 2016), a problem that has been already recognized in schizophrenia (Frith, 1992) and more recently in fronto-striatal disorders (Modinos et al., 2009).

The term “social cognition” embraces several subdomains including the ability of recognizing what others feel, think or do from facial expression, prosody or body posture (Uekermann et al., 2010). Social cognition regards how people make sense of other people and themselves. It focuses on how ordinary people think and feel about people and on how they think and feel about people (Fiske and Taylor, 2013). A core component of “social cognition” is the concept of Theory of Mind (ToM), which refers to the ability to infer other people’s mental states in terms of beliefs, desires or intentions (Premack and Woodruff, 1978; Baron-Cohen et al., 1985). Although this concept has been generally treated as a unitary process, some authors have argued for a distinction between “cognitive” and “affective” ToM (Shamay-Tsoory and Aharon-Peretz, 2007), depending on specific task demands. Briefly, cognitive ToM corresponds to the knowledge about others’ beliefs or intentions (Brothers and Ring, 1992; Coricelli, 2005), whereas affective aspects of ToM refer to the appreciation of the others’ emotional states (Brothers and Ring, 1992). This distinction has also been supported by imaging studies providing evidence that affective ToM is mostly impaired by ventromedial frontal lobe damage, whereas cognitive ToM is reduced by extensive prefrontal lesions (Hynes et al., 2006; Vollm et al., 2006; Shamay-Tsoory and Aharon-Peretz, 2007). In order to shed light on the complex mechanisms underlying ToM ability, some studies tried to deepen the relationship between ToM and cognitive abilities, focusing in particular on executive functions (EF) in normal population (Carlson et al., 2002; Ahmed and Stephen Miller, 2011). Circumstantial evidence for a link between EF and ToM comes from the fact that they both share a common developmental timetable, developing mainly in the preschool period (Reed et al., 1984; Zelazo et al., 1997), and that both sets of skills appear to be subserved by the prefrontal cortex in adults (Frith and Frith, 1999; Sabbagh and Taylor, 2000). Anyway, results in the literature are not univocal. In fact it is important to note that, in adult population, ToM can be assessed using different tests: Reading the Mind in the Eyes test (RMET) (Baron-Cohen et al., 2001), Strange Stories test (Happé, 1994), Faux Pas test (Gregory et al., 2002). Each test utilizes different cognitive mechanisms, implying that different cognitive processes are associated with different domains of ToM. For example, none of the EF variables was related to RMET performance (Ahmed and Stephen Miller, 2011). Finally, Fine et al. (2001) reported a case study of a patient suffering from amygdala damage who showed to be severely impaired in ToM, but did not show any impairment in his EF abilities (Fine et al., 2001).

Investigating affective ToM in HD proved to be useful for explaining why patients with HD can experience social difficulties and interpersonal problems, such as changes in empathy (Snowden et al., 2003). One popular test to assess affective ToM ability is the RMET (Baron-Cohen et al., 2001). In this test, the participant is presented with a series of 25 photographs of the eye region of different actors and actresses, and is asked to choose which of two words best describes what the person in the photograph is thinking or feeling. The “affective” ToM, as tested by means of the RMET, is mainly related to social and perceptive components, including the capacities involved in making fast empathic mental and evaluative judgments about people and their actions. Two studies (Allain et al., 2011; Eddy et al., 2012) investigated the “affective” aspect of ToM in patients with HD by means of the RMET test. Results showed consistent difficulties in inferring complex mental states from photographs of people even in the early stage of HD. However, these studies were inconsistent in disclosing whether cognitive impairments in HD population might have contributed to poor performance on the RMET test. Indeed the authors found that low scores on the RMET were associated with poor scores on verbal fluency task (Allain et al., 2011; Eddy et al., 2012) and on the Stroop test (Allain et al., 2011), whereas general cognitive function, assessed using the Mini Mental State Examination (MMSE) (Folstein et al., 1975) and the Mattis dementia rating scale (MDRS) (Mattis, 1976), did not apparently influence RMET performance (Allain et al., 2011). Such discrepancies might be related to the fact that HD population recruited for the latter study was early in the course of the disease and it is largely accepted that in HD cognitive impairments are selective in preclinical and early stages (with deficits in attention and executive function) and become more widespread in the later stages of the disease (Papoutsi et al., 2014).

The Montreal Cognitive Assessment (MoCA, Nasreddine et al., 2005) is a brief screening instrument for dementia that is thought to assess a broader array of cognitive domains (e.g., attention/executive functioning, visuospatial abilities and language) compared to other screening instruments. MoCA has been demonstrated to be able to detect cognitive impairment across a wide range of severity in HD, and to be even more sensitive than MMSE (Folstein et al., 1975) for detecting mild to moderate cognitive impairment in HD population (Videnovic et al., 2010), suggesting it is a useful screening measure of cognitive performance in HD (Videnovic et al., 2010; Gluhm et al., 2013).

The aim of the present study was, first of all, to deepen our knowledge about the ability of affective Tom in HD patients, particularly when they are in mild to moderate stages of the disease, in order to observe if any difference was present in ToM performance in comparison with patients in the early stage of the disease (Allain et al., 2011). Then, we aimed to examine if affective ToM was correlated to the global cognitive functioning, in order to investigate whether affective ToM task might represent a valuable instrument for studying social cognition in HD population independently from global cognitive dysfunction. To this aim, the MoCA questionnaire and the RMET were administered to HD patients in mild to moderate stages of the disease.

## Materials and Methods

### Participants

Fifteen patients (eight males, seven females) with HD were recruited from the outpatient Movement Disorders Clinic of the University of Genoa. The diagnosis of HD had been confirmed by genetic testing. Seven patients were taking fixed doses of psychiatric medication to address mood changes and/or anxiety and 13 were treated with medication to reduce choreic symptoms. At the time of the assessment, patients’ mean age was 53.6 years (range: 30–62 years, *SD* = ±9.6), level of education ranged from 5 to 18 years of schooling (mean: 11.86; *SD* = ±3.6) and the mean illness duration was 8.1 years (range: 1–20 years, *SD* = ±5.7). Regarding the severity of the disease, nine patients were in the mild whilst six patients were in the moderate stage, according to the Total Functional Capacity Scale. Patients were excluded in the presence of past history of neurological conditions other than HD, and severe visual impairments. The control group (HS) consisted of six males and nine females. Their mean age was 60.4 years (*SD* = ± 10.1; Range: 35–70), level of education ranged from 7 to 18 years of schooling (mean: 14.4; *SD* = ±3.9). In the HS group, during the recruitment phase, the following exclusion criteria were applied: (1) history of psychiatric and neurological disorders, (2) major depression, diagnosed by means of DSM IV criteria; and (3) general intellectual impairment (defined by means of MMSE age- and education- adjusted score < 24). Demographic and clinical features of HD and HS are summarized in **Table 1**.

**Table 1:** Demographic and clinical characteristics.

	HD (*n* = 15)	HS (*n* = 15)	Chi-square
			
	Mean ±*SD*	Mean ±*SD*	
Age (years)	53.6 ± 9.6	60.4 ± 10.1	χ^2^: *p* = 0.10
Education (years)	11.86 ± 3.6	14.4 ± 3.9	χ^2^: *p* = 0.13
Disease duration (years)	8.1 ± 5.7	–	–
UHDRS motor score	36 ± 19.5	–	–
TFC score	10.9 ± 2.15	–	–


*HD, Huntington Disease; HS, healthy subjects; UHDRS, Unified Huntington Disease Rating Scale; TFC, Total Functional Capacity.*

### Procedure

Participants were naïve to the purpose of the experiment and gave written informed consent after reading information leaflets about the study. Informed consent was obtained according to a procedure approved by the local ethics committee (Comitato Etico Regione Liguria, IRCCS Azienda Ospedaliera Universitaria San Martino—IST, Genoa, Italy) and to the Declaration of Helsinki. All participants underwent a neuropsychological battery of test and only HD patients were evaluated on their motor and general abilities (**Table 2**).

**Table 2:** Measures collected in HD and HS samples.

	HD	HS
RMET	+	+
UHDRS Motor Evaluation	+	-
TFC	+	-
MoCA	+	+
BDI-II	+	+


*HD, Huntington Disease; HS, healthy subjects; RMET, Reading the mind in the eyes test; UHDRS, Unified Huntington Disease Rating Scale; TFC, Total Functional Capacity; MoCA, Montreal Cognitive Assessment; BDI-II, Beck Depression Inventory II.*

#### Motor Evaluation

A movement disorder specialist examined the participants and rated the presence and severity of 15 individual motor signs using the UHDRS Motor Assessment (Huntington Study Group, 1996). The sum of motor signs can range from 0 to 124 with higher scores indicating more impaired motor function.

#### Total Functional Capacity (TFC)

A standardized scale was administered to patients in order to assess their capacity to work, handle finances, perform domestic chores and self-care tasks, and live independently. This scale was used to stratify manifest HD subjects as mild (TFC = 10–13) or moderate (TFC = 7–9) (Gluhm et al., 2013).

#### ToM Task

**FIGURE 1:**
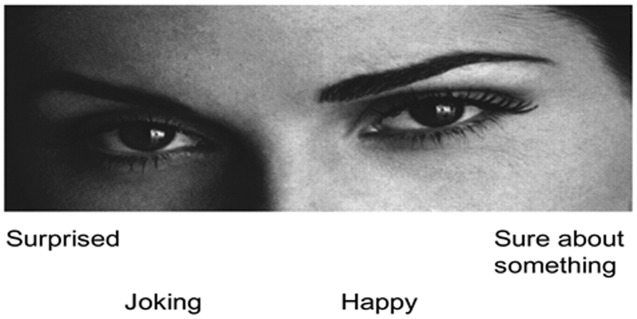
**Reading the mind in the eyes test (RMET) task stimulus example**.

The revised version of the RMET (Baron-Cohen et al., 2001) was used to assess the affective ToM ability. Participants were asked to imagine being inside the mind of the person shown in the photograph and subsequently attribute a mental state to them, trying to give a semantic definition of a possible mental state (“frightened” or “suspicious” for example). This process is assumed to involve an unconscious, automatic and rapid matching of past memories concerning similar expressions with a lexicon of mental state terms (Baron-Cohen et al., 2001). Specifically, the RMET task comprised a series of 36 photographs, of the eye region of a Caucasian actor (19 actors and 17 actresses). Presented in a random order, each photo had four possible state descriptors one at each corner and only one of these descriptors targeted the mental state depicted in the photo, while the others were foils (**Figure 1**). Participants were required to choose between these four descriptors which best described the eyes expressions of the actor in the photo. Participants could take all time they needed to choose the descriptor and continued to the following item only when ready. Performance was evaluated by measuring the percentage of correct responses. The RMET test is a simple but advanced ToM test and its internal consistency and test–retest stability were good for the Italian population (Vellante et al., 2013).

#### Cognitive Functioning

The MoCA, a brief 30-point test, was used to assess different cognitive domains. Individual items on the MoCA were divided into different cognitive-specific domains (attention, executive function, visuospatial abilities, language, memory, and orientation) based on previous research (Nasreddine et al., 2005).

The short-term memory recall task (5 points) involves two learning trials of five nouns and delayed recall after approximately 5 min. Visuospatial abilities are assessed using a clock-drawing task (3 points) and a three-dimensional cube copy (1 point). Multiple aspects of EF are assessed using an alternation task adapted from the Trail Making B task (1 point), a phonemic fluency task (1 point), and a two-item verbal abstraction task (2 points). Attention, concentration, and working memory are evaluated using a sustained attention task (target detection using tapping; 1 point), a serial subtraction task (3 points), and digits forward and backward (1 point each). Language is assessed using a three-item confrontation naming task with low-familiarity animals (lion, camel, rhinoceros; 3 points) and repetition of two syntactically complex sentences (2 points). Finally, orientation to time and place is evaluated (6 points). The MoCA proved to be an appropriate measure for cognitive screening taking into account different cognitive domains (Freitas et al., 2012).

#### Depression

To assess mood changes we used the Beck Depression Inventory II (BDI-II) (Beck et al., 1996), a 21 items self-report rating scale of depressive symptoms, such as irritability, low mood, lack of interest in enjoyable activities, and fatigue. The BDI-II is a relevant psychometric instrument, showing high reliability, capacity to discriminate between depressed and non-depressed subjects (Wang and Gorenstein, 2013).

### Statistical Analysis

Chi-square test was applied to assess gender differences between groups. Because of significantly non-normal distributions (according to the Shapiro–Wilk statistical test), non-parametric Mann–Whitney *U*-tests were used to analyze MoCA total scores, MoCA sub-scores in each of the cognitive-specific domains and RMET scores between groups (HD and HS). Regarding RMET, to observe if any difference was present in HD subjects between the two different stages of the disease, a further Mann–Whitney *U*-tests was performed. For data in which normality was assumed (BDI-II), a Student’s *t*-test for unpaired data was used to assess differences between groups.

To disentangle whether ToM ability could be associated with HD independently from years of education and affective status (BDI-II), the association between the ToM score and case–control status (HD and HS) was analyzed using logistic regression models. Then, the contribution of the cognitive functioning (total MoCA score) and of the six cognitive-specific domains (attention, executive function, visuospatial, language, memory, and orientation) was analyzed by running a Spearman correlation analysis between RMET score (expressed as percentage of correct responses), MoCA total score and MoCA sub-scores, separately for HD and HS groups.

A standard statistical package computed odds ratios (ORs), two-sided 95% confidence intervals (CIs) and *P*-values; *P* < 0.05 was considered to be significant.

All data analysis was performed using SPSS 22.0

## Results

Descriptive statistics for demographic and clinical data are reported in **Table 1**. HD and HS groups were matched for age, gender and education levels since no significant differences emerged between groups (*p* always > 0.05). In HD patients, TFC mean score was 10.91 (Range of 7–13) with nine subjects in the mild (TFC = 10–13) and six subjects in the moderate (TFC = 7–9) stages of the disease.

**FIGURE 2:**
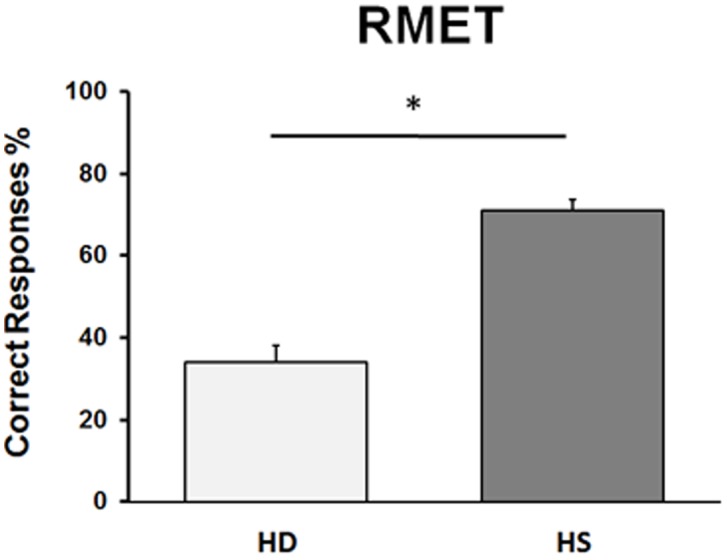
**Performance in the RMET in Huntington’s disease (HD) patients and in healthy subjects (HS).** On the ordinate, we report the % of correct responses. Bars indicate standard error means (SEM).

Statistical analysis on the percentage of correct responses at the RMET test revealed a significant difference between HD and HS groups (*U* = 9.50, *p* = 0.000001), showing that HD subjects performed significantly worse than HS (**Figure 2**). Regarding HD subjects, no difference between the two sub-groups of mild and moderate stages of the disease (*U* = 18, *p* = 0.29) was present.

Regarding MoCA (**Table 3**), HD patients performed significantly worse compared with HS. For total MoCA score, statistical analysis revealed a significant difference between groups (*U* = 3.50, *p* = 0.000006). The mean score for HD patients was 19.67 (±5.12 SD) with a range of 8–26, while for normal controls the average was 28.26 (±1.75 SD), ranging from 25 to 30.

**Table 3:** Neuropsychological assessment.

	HD (*n* = 15)	HS (*n* = 15)	Student’s *t*-test or Mann–Whitney *U*-test
		
	Mean ±*SD*	Mean ±*SD*	
BDI-II score	12.4 ± 11.5	5.6 ± 4.3	T: *p* = 0.041
MoCA Total score	19.87 ± 5.68	28.27 ± 1.75	U: *p* = 0.00014
MoCA Sub-score: Memory	1.6 ± 1.45	3.87 ± 1.36	U: *p* = 0.00066
MoCA Sub-score: Visuospatial abilities	2.47 ± 1.12	4 ± 0	U: *p* = 0.00011
MoCA Sub-score: Executive function	1.79 ± 1.39	3.73 ± 0.46	U: *p* = 0.00015
MoCA Sub-score: Attention	4.33 ± 1.68	5.93 ± 0.26	U: *p* = 0.0029
MoCA Sub-score: Language	4.13 ± 0.99	4.8 ± 0.41	U: *p* = 0.044
MoCA Sub-score: Orientation	5.13 ± 1.40	5.93 ± 0.26	U: *p* = 0.046


*HD, Huntington Disease; HS, healthy subjects; BDI-II, Beck Depression Inventory II; MoCA, Montreal Cognitive Assessment.*

The Mann–Whitney *U*-test showed that a significant difference was present between HS and HD in all the MoCA sub-scores (*p* always < 0.05) (**Table 3**).

Finally, regarding the specific measure of affective status (BDI-II), unpaired *t*-test showed a significant difference between HD patients and control subjects (*p* = 0.041).

Univariate logistic regression analysis showed a significant and positive association between ToM ability and presence of HD (**Table 4**). This association remained significant after having adjusted for years of education and BDI-II (**Table 4**).

**Table 4:** Logistic regression analysis of the association between ToM ability and HD.

HD versus HS	Wald	Exp(b)	*p*-value
RMET	7.40	2.54	*p* = 0.007
Adjusted for Education	6.19	2.99	*p* = 0.013
Adjusted for Education, BDI-II	5.38	2.80	*p* = 0.020


*HD, Huntington Disease; HS, healthy subjects, RMET, Reading the mind in the eyes test; BDI-II, Beck Depression Inventory II.*

**FIGURE 3:**
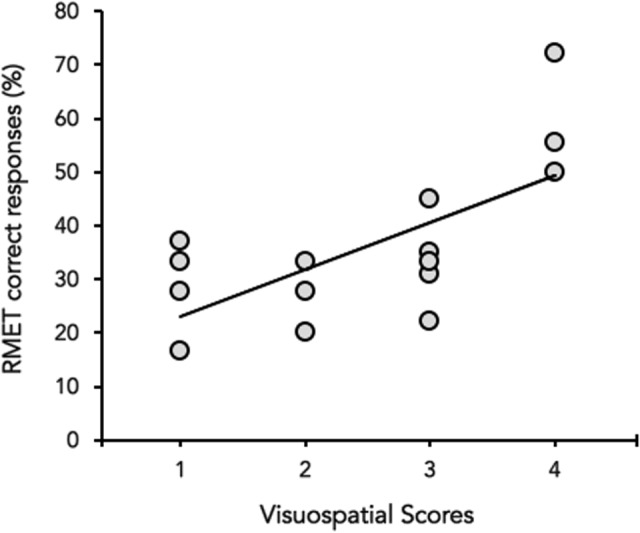
**Correlation between the visuospatial abilities score (*X*-axis) and the percentage (%) of correct responses of the RMET (*Y*-axis).** Data of Huntington’s disease participants in the mild to moderate stages of the disease are plotted together (gray circles).

Neither in HD and HS, a significant correlation emerged between MoCA (total score) and the percentage of correct responses at the RMET (HD: Spearman’s ρ = 0.3094, *p* = 0.26, HS: Spearman’s ρ = 0.1036, *p* = 0.24). However, when the correlation was performed between the percentage of correct responses at the RMET and each of the six sub-scores of MOCA separately, RMET performance significantly correlated with the visuospatial abilities score (Spearman’s ρ = 0.384, *p* = 0.018) in HD (**Figure 3**), whereas no significant correlation was found in HS (*p* always > 0.05).

## Discussion

The aim of this study was to assess whether the affective ToM ability (tested by means of the RMET) was impaired in the mild to moderate stages of HD, and whether there was an association between compromised ToM ability and the presence of cognitive impairment. We considered very important trying to further clarify this possible association, since data in the literature are conflicting (Allain et al., 2011; Eddy et al., 2014). In a disease characterized by cognitive decline, including memory and executive impairments and other psychiatric manifestations (Asselen et al., 2012), it becomes crucial to understand if an ability that is important for social interaction, such as affective ToM ability, is influenced or not by the cognitive impairment. However, it must be taken into account that our study is based on a small sample of subjects and therefore results should be read with caution.

To date, findings are consistent in showing that several facets of social cognition, including emotion recognition, are impaired in HD patients (Snowden et al., 2003). On this regard, it has been demonstrated that even preclinical and early HD patients had difficulties in recognizing emotional facial expression such as disgust, fear and anger (Johnson et al., 2007). Our results confirmed that HD patients in the mild to moderate stages of the disease poorly performed in affective ToM task, indicating that they had difficulties to infer other people’s emotional states. Furthermore, our results showed that affective ToM performance was not influenced by the depressive status even though there were significant differences in the BDI-II mean score between groups. Finally, in our sample of HD patients, ToM ability did not correlate with global cognitive functioning as assessed by MoCA total score, in accordance with the study of Allain et al. (2011). However, analyzing more in details MoCA sub-scores, the performance in RMET correlated with visuospatial abilities, but not with EF. This latter result seems therefore in contrast with a previous study in HD patients showing that executive dysfunction correlated with RMET thus suggesting that executive dysfunction could contribute to impaired affective ToM performance (Eddy et al., 2014). On the other hand, our findings are consistent with studies that dissociated the performance of affective ToM and executive tasks in patients with severe traumatic brain injury (Havet-Thomassin et al., 2006; Muller et al., 2010), in patients with frontal variant of frontotemporal dementia (Lough et al., 2001; Gregory et al., 2002), and even in a group of HD patients, early in the course of the disease (Allain et al., 2011). Indeed, cognitive rather than affective ToM seems to be mediated by dorsolateral fronto-striatal circuit and thus to be associated with executive dysfunction (Bodden et al., 2010a,b; Yu et al., 2012). Here, by analyzing MoCA total score and sub-scores, we found that only visuospatial abilities were associated to the performance in affective ToM task. One possible explanation for this result may deal with the nature of the RMET that requires an accurate scanning of the eye region in order to judge complex mental states from photographs of people’s eyes alone.

However, a recent study showed that symptomatic HD patients presented an emotion recognition deficit with a normal scanning pattern of the different regions of interest of the face (Asselen et al., 2012). Noteworthy, Asselen et al. (2012) assessed emotion recognition and eye movement patterns in a population of early HD patients and whether difficulties in face scanning may contribute to impaired emotion recognition in HD patients into mild to moderate stage of the disease has not been elucidated so far. One possible unifying explanation is that visuospatial deficits might contribute to low scoring at the RMET, even if affective ToM performances are more likely the results of higher order processing impairments (Sprengelmeyer et al., 1996).

Theory of Mind is thought to be mediated by a complex neuroanatomical network that includes the medial prefrontal cortex, the superior temporal sulcus region, the temporo-parietal junction, the temporal pole and the amygdala (Bodden et al., 2010a). Further, the rostral frontal regions, such as the orbitofrontal cortex send input to the striatum, giving rise to the “limbic” fronto-striatal circuit (Lanciego et al., 2012). On this basis, a contribution of basal ganglia to affective ToM ability has been hypothesized (Abu-Akel, 2003).

Both pathological (Vonsattel et al., 1985; Mann et al., 1993) and neuroimaging studies (Aylward et al., 1997) suggested that HD is characterized by a marked atrophy of the striatum (caudate and putamen). However, a recent imaging study showed that the various clinical features observed in HD were related to regionally specific lesions of cortico-basal ganglia networks rather than to the striatum only (Delmaire et al., 2013). Particularly, apathy correlated with structural abnormalities of the orbitofrontal cortex that is interconnected with the limbic circuit of the basal ganglia. In this scenario, we can hypothesize that affective ToM impairment in HD could implicate a selective dysfunction in the limbic (affective) cortico-basal ganglia-cortical circuit, and not in the associative one. If this is the case, testing affective ToM by means of RMET could be a useful tool to assess social cognition even when the disease progresses and cognitive dysfunction becomes more relevant.

Some limitations of this study deserve to be considered. The first concerns the relatively small sample size of our HD group. The lack of association of affective ToM with global cognitive functioning (in particular with EF) needs to be replicated with a larger number of participants in order to strengthen our findings. Further, the relationship between deficits in visuospatial abilities and impaired affective ToM performance might be explored in future studies with more specific tests, in order to better disclose their potential connection with affective ToM ability. The hypothesis related to the involvement of limbic basal ganglia circuit as neuroanatomical substrate of affective ToM impairment in HD should be confirmed through imaging studies. Finally, our research did not take into account the potential complex interrelationships between neuropsychiatric symptoms (such as irritability, aggression, and compulsive behaviors) (Van den Stock et al., 2015; Eddy et al., 2016) and compromised affective ToM. However, whether affective impairment, related to ToM, is a major component of neuropsychiatric manifestations in HD should be the subject of specific studies.

## Conclusion

Our study showed that using RMET to assess affective ToM ability could be a useful tool to evaluate HD patients who are in different stages of the disease, independently from their cognitive status. On this basis, affective ToM in HD might be instrumental for explaining why patients with HD can experience social difficulties and interpersonal problems, and, as a result, in developing psycho-educational interventions. The lack of relationship between affective ToM ability and global cognitive function should be considered for a better management of the different symptoms of the disease. In particular, when referring to patients with social difficulties and interpersonal problems we should consider that these symptoms are not influenced by patients’ cognitive functioning. The social impairment should be considered as a specific objective to work on, organizing patients’ management and therapeutic intervention.

## Author Contributions

ARDF, LA, GL, and EP: Conceived and designed the experiments. ARDF, GL, and RM: Performed the experiments. RM, ARDF, and LA: Analyzed the data. GL, LA, EP, ARDF, and GA: Wrote the paper. RM, PM, CS, and EP: Interpreted the data. LA, RM, PM, and CS: Drafted the article. GL, LA, EP, GA, and CS: Critically revised the article for important intellectual content.

## Conflict of Interest Statement

The authors declare that the research was conducted in the absence of any commercial or financial relationships that could be construed as a potential conflict of interest.
